# Adventitious Rooting in *Populus* Species: Update and Perspectives

**DOI:** 10.3389/fpls.2021.668837

**Published:** 2021-05-20

**Authors:** Florencia Bannoud, Catherine Bellini

**Affiliations:** ^1^Umeå Plant Science Centre, Department of Plant Physiology, Umeå University, Umeå, Sweden; ^2^Institut Jean-Pierre Bourgin, INRAE, AgroParisTech, Université Paris-Saclay, Versailles, France

**Keywords:** *Populus*, adventitious rooting, vegetative propagation, endogenous factors, environmental factors

## Abstract

*Populus* spp. are among the most economically important species worldwide. These trees are used not only for wood and fiber production, but also in the rehabilitation of degraded lands. Since they are clonally propagated, the ability of stem cuttings to form adventitious roots is a critical point for plant establishment and survival in the field, and consequently for the forest industry. Adventitious rooting in different *Populus* clones has been an agronomic trait targeted in breeding programs for many years, and many factors have been identified that affect this quantitative trait. A huge variation in the rooting capacity has been observed among the species in the *Populus* genus, and the responses to some of the factors affecting this trait have been shown to be genotype-dependent. This review analyses similarities and differences between results obtained from studies examining the role of internal and external factors affecting rooting of *Populus* species cuttings. Since rooting is the most important requirement for stand establishment in clonally propagated species, understanding the physiological and genetic mechanisms that promote this trait is essential for successful commercial deployment.

## Introduction

Adventitious roots (ARs) are formed post-embryonically from new cells of non-root tissues such as leaves and stems. Many species can produce ARs during normal development, but also in response to environmental stresses such as wounding, flooding, or nutrient deficiency (Steffens and Rasmussen, 2016). The ability to produce ARs is an essential step in clonal propagation and, ecologically, provides a selective advantage to plants with this type of propagation. In woody species propagation, using cuttings is economically important to amplify elite clones for plantations rapidly. ARs also play an important ecological role since they contribute to the survival of plants exposed to biotic and abiotic stresses (Steffens and Rasmussen, 2016), as well as to the dynamics of plant populations (Kinsman, 1990); and they also enhance the efficiency of phytoextraction of contaminated soils (Low et al., 2011). Moreover, a strong root system is essential for providing an adequate anchor, especially important in windbreaks (Zalesny and Zalesny, 2009).

The genus *Populus* is among the most economically important woody plants (FAO, 2014). It comprises about 30 species and is divided into six sections (Table 1), including poplars, cottonwoods, and aspens, with a worldwide distribution but mostly found in the Northern hemisphere (Dickmann and Kuzovkina, 2003; Lin et al., 2018). Most poplars rapidly produce ARs on hardwood cuttings, which makes them easy to propagate vegetatively by directly planting the cuttings in the field and so they have been extensively used in plantations throughout the world (Dickmann, 2006).

**TABLE 1:** Proposed taxonomic classification of the genus *Populus* and the rooting ability of each entry according to Dickmann and Kuzovkina (2003).

Section	Taxon	English common name	Notes and synonym	Rooting ability of hardwood cuttings
Abaso	*P. mexicana* Wesmael	Yaqui cottonwood	Monotypic section	Unknown

Turanga (Afro-Asian poplars)	*P. euphratica* Olivier	Euphrates poplar	Includes *P. diversifolia*	Variable
	*P. ilicifolia* (Engler) Rouleau	Kenyan poplar	Formerly synonymous with *P. euphratica*	
	*P. pruinosa* Schrenk	Desert poplar	Formerly synonymous with *P. euphratica*	

Leucoides (Swamp poplars)	*P. glauca* Haines	Asian swamp cottonwood	Formerly *P. wilsonii*	Poor
	*P. heterophylla* Linnaeus	Swamp cottonwood		
	*P. lasiocarpa* Oliver	Heart-leaf poplar		

Aigeiros (Cottonwoods, black poplar)	*P. deltoides Marshall*	Eastern cottonwood	Includes *P. sargentii*, *P. palmeri* and *P.* wislizenii	Generally good
	*P. fremontii S. Watson*	Fremont cottonwood	Includes *P. arizonica*	
	*P. nigra Linnaeus*	Black poplar		

Tacamahaca (Balsam poplars)	*P. angustifolia* James	Narrowleaf cottonwood		Very good
	*P. balsamifera* Linnaeus	Balsam poplar	Formerly *P. tacamahaca*	
	*P. cathayana* Rehder	Cathay poplar	May be synonymous with *P. suaveolens*; includes *P. purdomii*	
	*P. ciliata* Royle	Himalayan poplar	Heretofore in section Leucoides; the former *P. tristis* may be a hybrid with this species	
	*P. koreana* Rehder	Korean poplar	Probably synonymous with *P. suaveolens* or *P. maximowiczii*	
	*P. laurifolia* Ledebour	Laurel poplar		
	*P. maximowiczii* Henry	Japanese poplar	May be synonymous with *P. suaveolens*; includes *P. ussuriensis*	
	*P. simonii* Carrière	Simon poplar	Includes *P. przewalskii* and *P. kangdingensis*	
	*P. suaveolens* Fischer	Siberian poplar		
	*P. szechuanica*	Schneider Szechuan poplar		
	*P. trichocarpa* Torrey & Gray	Black cottonwood	May be synonymous with *P. balsamifera*	
	*P. yunnanensis* Dode	Yunnan poplar		

Populus (White poplars and aspens)	*P. alba* Linnaeus	White poplar		Variable for white poplars Aspens cuttings do not root
	*P. guzmanantlensis Vazquez and Cuevas*	*Manantlán poplar*	*May be synonymous with P. simaroa*	
	*P. monticola* Brandegee	Baja poplar	Aka *P. brandegeei*; may be naturalized *P. alba* var. subintegerrima	
	*P. simaroa* Rzedowski	Balsas poplar		
	*P. adenopoda* Maximowicz	Chinese aspen		
	*P. gamblei* Haines	Himalayan aspen		
	*P. grandidentata* Michaux	Bigtooth aspen		
	*P. sieboldii* Miquel	Japanese aspen	Includes *P. jesoensis*	
	*P. tremula* Linnaeus	Common aspen	Includes *P. davidiana* and *P. rotundifolia*	
	*P. tremuloides* Michaux	Quaking aspen		

*Populus* species have a fast growth rate, and they adapt well to marginal soils. They have many potential end-uses such as biofuel, fiber, timber, bioremediation, and animal feed (Stettler et al., 1996). Hybrid poplars, which are exceptionally fast-growing trees, have been used in many phytoremediation projects where their ability to establish rapidly after planting is essential, along with their extensive root system and a large biomass production (Zalesny et al., 2007). Because of these agronomic characteristics, together with the availability of the genome sequence, a small genome size, ease of propagation in tissue culture and their suitability for efficient genetic transformation, *Populus* trees have emerged as an experimental model system for studies of tree species. During recent decades, the rooting ability of stem cuttings has been one of the main traits selected for in breeding programs. Huge efforts have been put into identifying the mechanism controlling this trait. Many studies related to the endogenous and environmental modulators of AR formation in *Populus* have been published over more than 60 years. In this review we discuss these factors and highlight the differences observed among the different *Populus* species.

## Adventitious Rooting in *Populus*: The Origin and Phases of Rooting Are Genotype-Dependent

In *Populus*, the root system is very complex. Roots can originate in different ways: from the radicle of a seed, from an existing root system, as in the case of suckers, or from a cutting or abscised branch. Clonal propagation of *Populus* trees relies mainly on the last situation, *i.e.*, the formation of adventitious roots from stem or branch cuttings. AR formation is a complex biological process that includes different phases. Cell dedifferentiation is often required for cells to acquire rooting competence and respond to signaling factors. The induction phase is then characterized by the activation of a cell cycle leading to the formation of a primordium, and cell division can be observed. This is followed by the activation of the root primordium and the formation of new tissues, and the last phase is the out-growth of the root primordium where the root elongates, and vascular connections are formed (Legué et al., 2014). ARs normally develop from cells neighboring vascular tissues (Bellini et al., 2014). In stem cuttings of the poplar *Populus trichocarpa*, AR primordia seem to emerge from the cambium cells and their immediate derivatives (Rigal et al., 2012).

In some *Populus* species, preformed dormant AR primordia already exist (Zalesny and Zalesny, 2009; Hartmann and Kester, 2014). This is the case for the hybrid poplar “NL895” (*Populus euramericana: P. deltoides* × *P. nigra*) in which cuttings contain preformed and newly formed AR primordia that co-exist (Zhang Y. et al., 2019). In some other genotypes, primordia are induced as a response to external stimuli or a callus is formed at the base of the cutting prior to the differentiation of root primordia (Lovel and White, 1986).

Like for other traits, AR formation is subject to a strong genotypic effect in *Populus* trees (Dickmann et al., 2001), and the rooting ability varies significantly between the different genotypes (Dickmann and Kuzovkina, 2003; Zalesny and Zalesny, 2009; Table 1). Hardwood stem cuttings from sections Tacamahaca and Aigeiros show a good to very good capacity to produce ARs, even though considerable clone-to-clone variation occurs, especially in terms of rooting vigor. In contrast, cuttings from species and hybrids of the section Populus normally do not produce ARs, with the exception of some genotypes of *Populus alba*. Therefore, aspens cannot be propagated from hardwood stem cuttings, whereas softwood cuttings may root after an expensive process that requires greenhouse misting facilities (Dickmann et al., 2001). This inability to form roots from hardwood cuttings limits the planting of aspens on a commercial scale.

Several studies described the variation in the rooting capacity observed among trees from the *Populus* genus. In *P. nigra* cuttings, primordia were formed 4 days after cutting and roots emerged on the eighth day, while *P. alba* and *P. tremula* cuttings developed AR primordia 10 days after being cut, but roots did not elongate in *P. tremula* (Güneş, 2000). Chen et al. (1994) reported that within the Populus section, cuttings from white poplar (*P. alba)* rooted 14 days after cutting, whereas the aspen (*P. davidiana*) did not produce roots at all. In another study, results showed that the variability in different rooting traits observed in 21 clones of *Populus* species was due to the combination of genetic variation between and within groups, as well as to the environmental conditions and the ability of each genotype to respond to the contrasting field conditions (Zalesny et al., 2005). Even though only species from sections Aigeiros and Tacamahaca, the most widely cultivated ones, were included in this study, it is evident that different genotypes respond in distinct ways to external factors during AR formation.

Breeders have partially overcome the problems of poor rooting by producing hybrids between the Tacamahaca poplars, which root well, and the difficult-to-root genotypes that present traits of interest. Nevertheless, not all hybrids exhibit the desired improved trait, and it is, therefore, essential to identify the physiological and genetic mechanisms that affect AR formation.

## Adventitious Rooting in *Populus* Is a Complex Trait

Adventitious rooting in *Populus* is a complex trait and several genetic studies have attempted to identify genome regions that contain genes controlling it. Many QTL have been detected for adventitious rooting-related traits, such as the total number of roots, the total root length, the average root diameter, the surface root area, and the root volume (Zhang et al., 2009; Ribeiro et al., 2016; Sun et al., 2019). For these traits, medium to high heritabilities were observed suggesting that adventitious rooting is under a strong genetic control and that major QTL can be detected. Different biparental populations were used for these mapping studies and this is probably the main reason why no overlap between regions with significant QTL have been identified among them. All three mapping populations examined so far have been obtained by crossing species from sections Aigeiros and Tacamahaca, cuttings from both of which exhibit good rooting performance. In order to assess most of the genetic variation between parents and obtain QTL that explain a high proportion of the phenotypic differences observed, it is important to obtain a segregating population using as a parent one of the difficult-to-root aspens, such *as P. tremula*. Furthermore, the use of different markers, software and mapping models may contribute to the different results obtained in the studies. Despite this, several QTL have been identified confirming that adventitious rooting in *Populus* is a quantitative trait controlled by distinct regions, and differences between genetic backgrounds are expected.

Moreover, transcriptomic analyses have also been performed to identify candidate genes related to adventitious rooting in *Populus*. RNA from either the base of the stem cuttings at different AR developmental phases, or from fully developed roots have been collected (Table 2). The genetic localization and further identification of differentially expressed genes have proved a successful way to identify candidate genes in other species for different traits and, therefore, transcriptomic analyses represent a good complementary approach to identify genes involved in AR formation in *Populus*. The analysis of transcriptomes from different individuals that exhibit good and poor rooting could be performed to identify differentially expressed genes located within regions associated with ARs, obtained from either QTL or genome wide association. In the study by Sun et al. (2019), RNA-seq for easy- and difficult-to-root *P. deltoides* genotypes was carried out and some differentially expressed genes were identified within the QTL boundaries. Nevertheless, the RNA used in this study was collected from young elongated adventitious roots and, therefore, no differentially expressed genes could be identified during the initial phases of AR formation. It has been suggested that differences in gene transcript level occur during the first 24 h after cutting since most of the genes change their expression pattern during this period (Ramirez-Carvajal et al., 2009). Therefore, it is likely that the early responses that initiate adventitious roots in *Populus* species happen during the very first hours after wounding. Selecting good rooting clones at an early stage of plant development is a desirable step in breeding programs, therefore, it would be of a great value to identify genes that could serve as markers to characterize the rooting ability of a clone early on in tree production.

**TABLE 2:** Transcriptomic studies performed in different *Populus* species in order to identify candidate genes for adventitious root formation.

Species used	Type of technology used	AR stage	RNA collected from	Culture	References
Hybrid: *P. deltoides* × *P. euramerican*a	RNA-seq	AR outgrowth	Emerged ARs	*In vitro* tissue culture	(Xiao et al., 2020)

*P. ussuriensis*	RNA-seq	Induction, activation and outgrowth	Stem base at 0, 0.5, 1, 2, 6, 12, 24, 48, and 96 h after cutting	*In vitro* tissue culture	(Wei et al., 2020)

Hybrid Clone 84K: *P. alba* × *P. glandulosa*	RNA-seq	Induction	Stem base at 0, 12, 24, and 48 h after cutting	*In vitro* tissue culture	(Shu et al., 2019)

Hybrid: *P. deltoides* × *P. simonii* and parental lines	RNA-seq	AR outgrowth	Emerged ARs from genotypes with different rooting ability	Hydroponic culture in greenhouse	(Sun et al., 2019)

Hybrid: *P. deltoides* × *P. euramerican*a	RNA-seq	Induction, activation and outgrowth	Stem base at 0, 48, 96, 144, and 192 h after cutting	*In vitro* tissue culture	(Zhang Y. et al., 2019)

Hybrid: *P. trichocarpa* × *P. deltoids*	Microarray	Induction, activation and outgrowth	Stem base at 0, 24, 48, 96, and 192 h after cutting	Hydroponic culture in greenhouse	(Ribeiro et al., 2016)

*P. trichocarpa*	Microarray	Induction, activation and outgrowth	Stem base at different stages according to microscopic observations	Hydroponic culture in greenhouse	(Rigal et al., 2012)

Hybrid: *P. tremula* × *P. alba*	Microarray	Induction	Stem base at 0, 6, 24, and 48 h after cutting	Potting soil in greenhouse	(Ramirez-Carvajal et al., 2009)

## Endogenous Factors Influencing Rooting of Cuttings

An early response to wounding will induce rooting in the cuttings, and this depends on other factors besides the genetic background of the plant. Endogenous stimuli such as carbohydrate and mineral nutrition, phytohormones and other biochemical compounds will also affect the rooting performance of cuttings.

### Status of the Mother Plants

#### Physiological Condition, Size and Position of the Cuttings, and Date of Shoot Collection

The physiological and biochemical quality of the mother plants affects the rooting ability of cuttings. After the cutting is separated from the stock plant, the influx of nutrients is disturbed and, therefore, the survival of the plant relies on the availability of mineral elements within the cutting until the nutrient uptake capacity, *i.e*., the rooting system, is restored. This initial stock of nutrients is determined by the physiological/nutritional status of the mother plants. Carbohydrates are the main source of energy during AR formation in cuttings (Veierskov, 1988; Druege et al., 2004; Ruedell et al., 2013). Veierskov (1988) concluded that, when the other physiological conditions were favorable, a good carbohydrate stock improved the success of rooting. In pelargonium (*Pelargonium* × *hortorum*) cuttings, both initial carbohydrate and nitrogen content can limit root formation (Druege et al., 2004). Moreover, carbohydrate distribution and allocation in the cuttings can be even more important than the total content (Ruedell et al., 2013). In the case of woody species such as the easy-to-root *Eucalyptus saligna* (da Rocha Corrêa et al., 2005), *Pinus radiata* (Li and Leung, 2000), or the apple tree (*Malus domestica*) (Calamar and De Klerk, 2002), the addition of sugars to the growing media during the induction phase has a positive effect on rooting. In all these studies, an interaction between carbohydrates and the phytohormone auxin was observed. Mishra et al. (2009) showed that in *Arabidopsis thaliana*, auxin homeostasis and signaling during root development were modulated by increasing concentration of exogenously applied glucose. Later, Ahkami et al. (2013) proposed a model that integrates the relationship between auxin, polar auxin transport, primary metabolism and AR formation in leafy stem cuttings of petunia (*Petunia hybrida)*. In the difficult-to-root hazelnut (*Corylus avellana*), rooting was positively correlated to the carbohydrate content in stem cuttings which was positively influenced by leaf photosynthesis activity (Tombesi et al., 2015).

Carbohydrate content is related to the size of the cutting and to the location of the cutting on the mother plant, but is also affected by seasonal variation. Many studies have tried to identify the best size and position of the cutting, as well as the time of the year when it should be collected in order to improve rooting in *Populus* species (Allen and McComb, 1956; Dickmann et al., 1980; Phipps and Netzer, 1981; Schroeder and Walker, 1990; Houle and Babeux, 1993; Desrochers and Thomas, 2003; Zalesny et al., 2003). It is generally accepted that smaller cuttings obtained from the tip portion of a shoot produce fewer ARs than those from the basal portion. Nevertheless, inconsistencies between studies were found, especially when different clones were tested. According to Dickmann et al. (1980), larger diameter cuttings survived better and produced taller shoots in three clones of *Populus*, but Bogdanov (1965) observed a wide variation among the clones relative to the cutting diameter. Desrochers and Thomas (2003) showed that the diameter of the cuttings from four different poplar clones was not a critical variable for rooting, whereas the length of the cutting did influence the rooting success. Ten-centimeter cuttings showed a higher percentage of rooting compared to five-centimeter cuttings, but since there was no difference in the root dry mass between the two cutting lengths, longer cuttings produced plants with smaller root/shoot ratios (Desrochers and Thomas, 2003). It was suggested that longer and larger cuttings have more absolute carbohydrate reserves, which would explain their better rooting ability. Nevertheless, some studies have rejected this hypothesis, since carbohydrate reserves are not the only factors to be considered. In fact, cuttings from the difficult-to-root *Populus tremula* failed to translocate assimilates to the bottom of the cuttings and this was probably the reason for their inability to form roots rather than insufficient carbohydrate reserves (Okoro and Grace, 1976). Cutting diameter is related to the original position on the mother plant. Those cuttings originating from the base of the stools, which are normally bigger, have higher rooting rates than the ones coming from the top (Schroeder and Walker, 1990; Desrochers and Thomas, 2003). In a study of 21 *Populus* clones from different genomic groups, it was observed that cuttings originating from the basal third of the shoot system of the stool plant exhibited greater rooting-related traits, such as root dry mass, number of roots and total root length, than those from the apical and middle positions (Zalesny et al., 2003), and the differences observed were genotype-dependent (Zalesny and Wiese, 2006). Similar results were obtained in four different stone fruits and the difficult to root *Lobostemon fruticosus* where cuttings collected from the basal part presented better rooting performance than those collected from the apical part of the plant (Swarts et al., 2018; Tsafouros et al., 2019).

Another critical factor influencing rooting of *Populus* cuttings is the time of the year when cuttings are collected from the mother plants. A chilling period is required for parental shoots to break dormancy and achieve good rooting and growth. However, if shoots are collected too late in the dormant season, reserves are translocated to buds and used for aboveground growth which reduces the potential to form ARs (Zalesny and Zalesny, 2009). This is in accordance with the fact that in cuttings from two hybrid poplars, the highest sugar content was found in early winter, and it declined through early spring (Fege and Brown, 1984). In the study by Zalesny and Wiese (2006), wide variation was observed in the number of roots and root dry weight of cuttings from different *Populus* clones when collected at different time points, with a trend of increasing roots when shoots were collected after February in the Northern hemisphere. These results agreed with those of Houle and Babeux (1993) who suggested that, in order to optimize the rooting ability of cuttings and minimize differences between clones, *Populus* cuttings should be sampled early in the season before bud break or shortly thereafter. Results of these studies show that even though AR formation based on time of sampling is genotype-dependent, better results are obtained when cuttings are taken after a period of chilling and before the early spring when the carbohydrate content is relocated.

#### Aging of the Mother Plant

During development, plants undergo distinct phases which comprise a period of vegetative growth with juvenile and mature phases, and a reproductive phase, which may be followed eventually by seed set and senescence (Huijser and Schmid, 2011). In trees, morphological and physiological changes, such as shoot height and diameter, leaf shape, stomatal conductance, photosynthesis and respiration rates, and the decrease in rooting competence, are observed during these phases (Díaz-Sala, 2014). Since the ability to improve elite genotypes relies mainly on the rooting capacity of clones, the loss of rooting competence is one of the most important economic factors that limit propagation in tree species (Bellini et al., 2014). Regeneration efficiency is higher in plant tissues at earlier developmental stages and the age threshold at which this efficiency declines varies between species and clones. With aging and tree maturation, cells that form ARs lose competence for *de novo* regeneration of roots (Díaz-Sala, 2014). In the aspens *P. tremula* and *P. tremuloides*, for example, cuttings from mature trees fail to root but those from root suckers, with juvenile characteristics, can easily form roots (Heybroek and Visser, 1976). Moreover, in cottonwood (*P. deltoides*) the age of the cutting is associated with a significant decrease in the rooting ability (Allen and McComb, 1956).

Since auxin is one of the main hormones related to AR formation, several studies have focused on the differences in auxin content between young and mature tissues. It has been hypothesized that, compared to younger ones, mature tissues would respond more slowly or not at all to auxin. However, this hypothesis has been rejected, at least with pine (*Pinus*), for which the loss of ability to form ARs in mature tissues in response to auxin is not due to the lack of initial auxin responses, but rather to an intrinsic incapacity of cells to organize into a root meristem in response to auxin (Díaz-Sala et al., 1996; Hutchison et al., 1999; Greenwood et al., 2001; Busov et al., 2004). In *Eucalyptus globulus*, where age negatively affects rooting capacity, it has been found that even though the endogenous auxin content and sensitivity are decreased in older cutting donor plants, the detrimental effect of age is due to a combination of many other factors (Aumond et al., 2017).

It is often assumed that micropropagation and tissue culture rejuvenate the plants and restore rooting ability, especially in some difficult-to-root species (Gupta et al., 1981). Haapala et al. (2004) showed that cuttings from the hybrid aspen *P. tremula* × *P. tremuloides*, which is a difficult-to-root genotype, could root easily after being rejuvenated through micropropagation *in vitro*. Even though the negative effect of maturation tends to be less for *Populus* compared to other forest tree species, differences between the taxonomic groups within the genus do exist (Jansson et al., 2010). It is, therefore, important to identify the genetic and molecular factors that influence these differences between juvenile and mature cuttings in terms of rooting ability, especially for the difficult-to-root genotypes like the aspens, in which hardwood cuttings do not normally root, but softwood cuttings are able form adventitious roots.

### Phytohormones

**FIGURE 1:**
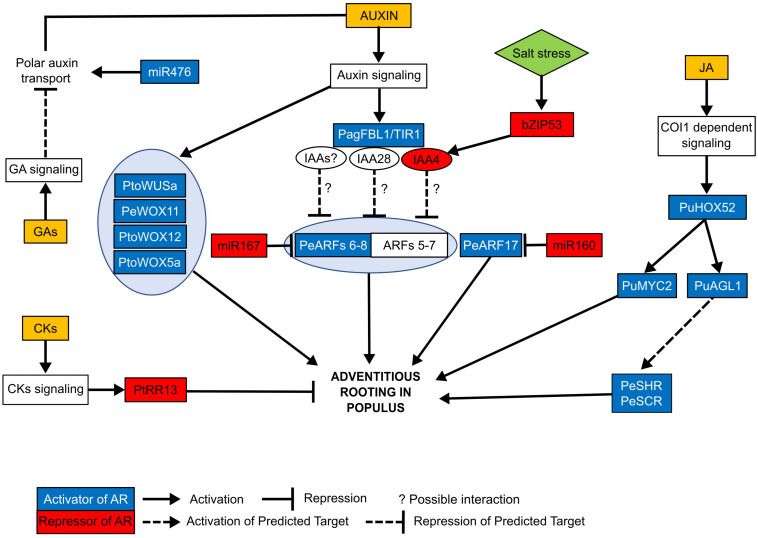
Summary of the molecular pathways controlling adventitious rooting in different *Populus* species. Positive (highlighted in blue) and negative (highlighted in red) regulators of adventitious rooting have been identified in different *Populus* species. The *TIR1* homolog *PagFBL1* interacts with IAA28 protein. This interaction might release the activity *AUXIN RESPONSE FACTORS (ARF) 7* and *5* and promote adventitious rooting. Another *AUX/IAA* gene, *IAA4*, was shown to inhibit AR formation. Salt stress induces the expression of transcription factor *bZIP53*, which binds to the promoter region of *IAA4* activating its expression and repressing AR formation, probably through the regulation of *ARFs*. Additional *AUX/IAA* might also regulate adventitious rooting in *Populus* through the *ARFs* as it has been described in arabidopsis where multiple IAAs interact with different *ARFs*. *ARFs 8* and *17* have been identified to promote rooting in *Populus*, and their transcriptional activity is negatively regulated by micro-RNAs, *miR167* and *miR160* respectively. Another miRNA, *miR476a*, has been very recently identified to positively regulate AR formation in *Populus* through a mitochondria-dependent pathway which activates the auxin polar transport. *WUSCHEL-related Homeobox* (*WOX5a*, *11*, *12*, and *WUSa*) genes promote rooting during the first stages of AR formation. Cytokinins (CKs) and giberellins (GA) negatively regulate adventitious rooting. CKs repress AR formation through the CK typeB response regulator, *PtRR13*, whereas gibberellins signaling inhibits rooting probably through the disturbance of polar auxin transport. In *Populus*, jasmonate (JA) has been recently shown to promote adventitious rooting through the COI1-dependent signaling. Transcription factor *PuHOX52* is induced by the addition of JA and promotes adventitious rooting. *PuMYC2* is a target of *PuHOX52* and a transcriptional activator of JA signaling, which acts as a positive regulator of AR development in *Populus*. MADS-box transcription factor (*PuAGL1*), also promotes rooting downstream the COI1-signaling and possibly targets *PuSHR* and *PuSCR* which were previously described to be involved in the regulation of adventitious rooting in *Populus*. The different *Populus* species abbreviation where genes have been first identified are Pe: *Populus euramericana*, Pde: *Populus deltoides*, Pt: *Populus trichocarpa*, Pto: *Populus tomentosa*, Pu: *Populus ussuriensis*. A complete description of genes is described in Table 3.

Among endogenous modulators of adventitious rooting, phytohormones are the most important ones (Bellini et al., 2014). Multiple hormones regulate the complex process of AR formation, among which auxin seems to be the master regulator and the link of the hormonal crosstalk during this process (Druege et al., 2019; Lakehal and Bellini, 2019; Lakehal et al., 2020b). During the last decade, several genes involved in adventitious rooting have been identified in different *Populus* species (Table 3 and Figure 1) and several assays have been carried out to evaluate the physiological effect on AR of several hormones. Results showed that, even though a specific role on AR development is still unclear for some of the hormones, a complex interaction exists.

**TABLE 3:** List of genes identified to regulate adventitious rooting in different *Populus* species.

Gene name	Gene family	Populus species	Role on Adventitious rooting	Mode of action	References
*PagFBL1*	*TIR1* homolog	Clone 84: *P. alba* × *P. glandulosa*	Promotes AR primordia	Targets IAA28 to regulate AR primordia emergence	(Shu et al., 2019)

*PtAIL1*	*AP2/ERF* transcription factor	*P. trichocarpa*, Clone T89: *P. tremula* × *P.tremuloides*, and *P. tremula* × *P. alba*	Promotes the formation of root primordia in an early stage of adventitious rooting	Play a role in cell division activity	(Rigal et al., 2012)

*PeARF8.1/2*	*AUXIN RESPONSE FACTOR*	Clone T89: *P. tremula* × *P. tremuloides*	Promotes AR formation	Regulates the expression of auxin responsive genes	(Cai et al., 2019)

*miR167a*	Micro-RNA	*Clone T89 : P. tremula* × *P. tremuloides*	Inhibits adventitious rooting	Targets *PeARF6.1/2* and *PeARF8.1/2* and inhibits its transcripts	(Cai et al., 2019)

*PeARF17.1/2*	*AUXIN RESPONSE FACTOR*	*P. davidiana* × *P. bolleana*	Promotes AR formation	Regulates the expression of auxin responsive genes	(Liu et al., 2019)

*peu-miR160a*	Micro-RNA	*P. davidiana* × *P. bolleana*	Inhibits adventitious rooting	Targets *PeARF17.1*/*2* and inhibits its transcripts	(Liu et al., 2019)

*PuHOX52*	HD-Zip I transcription factor	*P. ussuriensis*	Positive regulator of adventitious rooting downstream of the COI1-dependent signaling	Targets *PuMYC2* and *PuAGL1* and activates its expression	(Wei et al., 2020)

*PuMYC2*	Transcriptional factor on jasmonate signaling	*P. ussuriensis*	Positive regulator of adventitious rooting downstream of the COI1-dependent signaling	Master regulator of JA signaling pathway	(Wei et al., 2020)

*PuAGL1*	*MADS* box transcription factor	*P. ussuriensis*	Positive regulator of adventitious rooting downstream of the COI1-dependent signaling	Regulates root meristem cell proliferation and targets *PuSHR* and *PuSCR*	(Wei et al., 2020)

*PtaERF003*	*AP2/ERF* transcription factor	Clone 84*: P. alba* × *P. glandulosa*	Promotes adventitious rooting	External auxin application induces *PtaERF003* expression	(Trupiano et al., 2013)

*Big Leaf (BL)*	*AP2*-domain transcription factor (ortholog of *STERILE APETALA*)	*P. tremula* × *P. alba*	Promotes adventitious rooting	*BL* might interact with *PtAIL1*	(Yordanov et al., 2017)

*bZIP53*	*bZIP* transcription factor	NL895 hybrid: *P. deltoides* × *P. euramericana, and Shanxinyang hybrid: P. davidiana* × *P. bolleana*	Inhibits AR growth	Binds to the promoter of IAA4-1/2 and activates its expression	(Zhang et al., 2020)

*IAA4*	Indole-3-acetic acid inducible	NL895 hybrid: *P. deltoides* × *P. euramericana*, and Shanxinyang hybrid: *P. davidiana* × *P. bolleana*	Inhibits AR growth	Might repress *ARF*s expression	(Zhang et al., 2020)

*PtRR13*	type-B cytokinin response regulator	*P. tremula* × *P. alba*	Acts downstream of CK to repress AR formation	Plays a negative role by activating *COV1* homolog which interferes with the vascular continuity between root primordia and stem vascular tissue. It also activates *Pleiotropic Drug Resistance transpoter9* (*PDR9*) perturbing the auxin gradient required for adventitious rooting, and inhibits ethylene-inducible expression of *TINY*-like transcription factors	(Ramirez-Carvajal et al., 2009)

*PtoWUSa*	*WUSCHE*L-related Homeobox transcription factor	*P. alba* × *P. glandulosa*	Promotes adventitious rooting at the induction phase	It could regulate polar auxin transport	(Li et al., 2020)

*PeWOX11a/b*	*WUSCHEL*-related Homeobox transcription factor	*P. euphratica* and *P. tomentosa*	Promotes adventitious rooting at the induction phase	Involved in the first step of the cell fate transition during root organogenesis in arabidopsis	(Xu et al., 2015)
	
*PtoWOX12*	*WUSCHEL*-related Homeobox transcription factor	*P. tomentosa*	Promotes adventitious rooting at the induction phase		(Liu et al., 2014a)

*PtoWOX5a*	*WUSCHEL*-related Homeobox transcription factor	*P. alba* × *P. glandulosa*	Promotes adventitious rooting. Expressed during callus formation and AR elongation	Might regulate *CYCD* genes which are involved in cell division and differentiation	(Li et al., 2018)

*GA20*	Gibberellin biosynthetic gene	*P. tremula* × *P. tremuloides*	Inhibits AR formation	Gibberellins inhibit AR and appears to perturb polar auxin transport	(Mauriat et al., 2014)

*GAI*	*GIBERELLINS INSENSITIVE*	*P. tremula* × *P. alba*	Promotes adventitious rooting	Represses gibberellin signaling	(Busov et al., 2006)

*PdeCML23-1*	*CALMODULIN*-like	NL895 hybrid: *P. deltoides* × *P. euramericana*	Promotes AR elongation	Modulates cytosolic Ca concentration by inhibiting the transport of Ca into the cytoplasm.	(Xiao et al., 2020)

*PeSHR*	*SHORT-ROOT* transcription factor	NL895 hybrid*: P. deltoides* × *P. euramericana*	Positive regulator of adventitious rooting	Key regulator of root radial patterning and stele cell specification and maintenance	(Xuan et al., 2014)
	
*PeSCR*	*SCARECREW* transcription factor				

*miR476a*	Micro-RNA	*P. tomentosa*	Positive regulator of adventitious rooting	Directs the cleavage of *RFL* (*Restorer of Fertility*) genes, which restrict mitochondrial energy production, resulting in an increase of the mitochondrial functionality. This dynamic regulation of mitochondrial homeostasis modulates the auxin pathway by the activation of the efflux auxin carriers	(Xu et al., 2021)

*PtHDT902*	*HISTONE DEACETYLASE*	Clone 84: *P. alba* × *P. glandulosa*	Inhibits AR formation	Regulates adventitious rooting possibly by upregulating gibberellin biosynthesis	(Ma et al., 2020)

*PtTDIF2* and *PtTDIFL2*	*TRACHEARY ELEMENT DIFFERENTIATION INHIBITORY FACTOR (TDIF and TDIF-like)*	*P. tremula* × *P. alba*	Promotes the formation of adventitious roots	Promotes adventitious rooting by activating the auxin signaling cascade	(Yue et al., 2020)

					

#### Auxin

Auxins are plant hormones involved in various physiological events such as apical dominance, vascular differentiation, cell expansion, floral bud development and lateral and adventitious root formation. In the latter, auxin is considered the master regulator in different species. High auxin concentration is required for AR induction during the first hours after cutting, but is inhibitory during the subsequent AR initiation and expression phases (da Costa et al., 2013; Druege et al., 2016).

External applications of auxin are used to promote and accelerate the rooting of cuttings in different species (Blythe et al., 2007). The most abundant natural auxin is Indole 3-acetic acid (IAA), whereas Indole-3-butyric acid (IBA) is the one generally used as an exogenous auxin for stimulating AR formation in most species, and more specially in difficult-to-root genotypes. IBA is more stable than IAA and is converted into IAA in the plant (Van der Krieken et al., 1993; De Klerk et al., 1999). In poplar, shoot cuttings grown *in vitro* in the presence of anti-auxin agents, IAA competitors or inhibitors of auxin polar transport, during the inductive phase of adventitious rooting caused complete inhibition of rooting (Bellamine et al., 1998), in contrast IBA treatment induced root primordia in white poplar (*Populus alba*) cuttings, increasing the number of ARs per cutting (Harfouche et al., 2007). Moreover, in the aspen *P. tremula*, in order to increase the endogenous auxin content, the *UGT* gene from corn, encoding a UDPG-transferase that catalyzes the conjugation of IAA with glucose for easier transport, was introduced. A correlation between the enzyme activity, the content of free IAA and root formation was found in this transgenic line, thus confirming the promoting role of auxin in AR formation in this difficult-to-root aspen (Salyaev et al., 2006). Several auxin biosynthetic and signaling pathway genes, including transcription factors, have been identified in arabidopsis and other species and have been shown to influence AR formation (reviewed in Bellini et al., 2014; Pacurar et al., 2014; Lakehal and Bellini, 2019). Auxin is perceived by the receptor SCF^TIR1/AFB2^ which, in the presence of auxin, binds to the AUXIN/INDOLE-3-ACETIC ACID INDUCIBLE (Aux/IAA) proteins which will be sent for degradation. In arabidopsis, it has been shown that auxin controls AR initiation by modulating jasmonate homeostasis. It acts through an Aux/IAA-ARF module including two positive regulators (auxin responsive factors *ARF6* and *ARF8*) whose transcriptional activity is regulated by at least three Aux/IAA genes (*IAA6*, *IAA9*, and *IAA17*), and one negative regulator *ARF17*. This module controls the expression of three *Gretchen Hagen* genes (*GH3.3*, *GH3.5*, and *GH3.6*) that encode acyl-acid-amino synthetases that inactivates IAA and jasmonic acid, a known AR inhibitor in arabidopsis hypocotyls (Gutierrez et al., 2009, 2012; Lakehal et al., 2019). In *Populus*, it has also been demonstrated that auxin-related genes play a key role in AR formation. The poplar homolog of the arabidopsis auxin receptor *TIR1*, *PagFBL1*, has been shown to regulate adventitious rooting and to interact with IAA28 in the hybrid *P. alba* × *P. glandulosa* (Shu et al., 2019). The overexpression of *PagFBL1* induced AR formation and increased root biomass, whereas its downregulation had the opposite effect. In the presence of exogenous auxin, *PagFBL1* interacted with PagIAA28, and transcriptomic data revealed that *PagARF5* and *7*, and *PagGH3* genes (*PagGH3.1*, *PagGH3.5*, and *PagGH3.6*) were upregulated during AR initiation, suggesting that the PagFBL1-PagIAA28 interaction may release *PagARF*s activity (Figure 1) and subsequently induce *GH3* genes to promote adventitious rooting. In arabidopsis hypocotyls it has been shown that *ARF7* act together with *ARF19* as positive AR regulators by activating the downstream transcription factors *ORGAN BOUNDARIES DOMAIN* (*LBD*) *LBD16* and *LBD18* (Lee et al., 2019). Recently, Zhang et al. (2020) showed that the overexpression of the poplar *bZIP53* transcription factor had a negative effect on AR development by controlling the expression of *IAA4-1* and *IAA4-2* (Figure 1). *bZIP53* was shown to bind to the promoter of *IAA4-1* and *IAA4-2* and activate their expression. In addition, the inducible over-expression of *IAA4-2* reduced the number of ARs in poplar, and this was antagonized by exogenously applied IAA, suggesting that the bZIP53*-*IAA4 regulatory module could be involved in the auxin signaling pathway. These results are in agreement with the model described in arabidopsis, where *AUX/IAA* act as negative regulators of adventitious rooting by repressing the transcriptional activity of *ARF* genes, suggesting conserved mechanisms between both species. AUX/IAA proteins have been shown to interact with each other and form oligomers that repress *ARF*s genes (Korasick et al., 2014), and regulate adventitious rooting (Lakehal et al., 2019). Therefore, it is likely that in *Populus* more Aux/IAAs interact to regulate ARF activity.

Micro-RNAs (miRNA) regulate several plant developmental processes, including AR formation as shown for the complex regulatory mechanism including *AtARF6, AtARF8*, and *AtARF17* and their respective regulatory miRNA, *miR167*, and *miR160*, which seems to be conserved in arabidopsis and *Populus* (Gutierrez et al., 2009; Cai et al., 2019; Liu et al., 2019). In the hybrid poplar (*P. tremula* × *P. tremuloides*), *ARF8* overexpression promoted adventitious rooting, whereas the overexpression of the miRNA *miR167*, which targets *ARF8* mRNA, negatively regulated rooting, as in arabidopsis (Gutierrez et al., 2009; Cai et al., 2019; Figure 1). Liu et al. (2019) demonstrated that *ARF17*, which is a negative regulator of adventitious rooting in arabidopsis (Gutierrez et al., 2009), was a positive regulator in the hybrid *P. davidiana* × *P. bolleana* (Figure 1). Its overexpression promoted AR formation, and the overexpression of *miR160*, which targets *ARF17* mRNA, inhibited AR initiation (Liu et al., 2019). These results suggest that the role of *ARF17* in the control of rooting might be species-dependent, while that of *ARF8* seems to be conserved. In addition, very recently, Xu et al. (2021) found that *miR476a* regulates wound-induce AR formation in *Populus tomentosa* by suppressing several *Restorer of Fertility* (*RFL*) genes, which restrict mitochondrial energy production, resulting in an increase of the mitochondrial functionality. This dynamic regulation of mitochondrial homeostasis modulates the auxin pathway by the activation of the auxin efflux carriers *PIN-FORMED2*/*5b* (*PIN2*/*5b*) (Figure 1), which are exclusively expressed during AR formation (Liu et al., 2014b), promoting adventitious rooting. The overexpression of *MIR476a* increased the number of ARs in stem cuttings. The *MIR476a-OE* lines presented a denser and shorter root system, with an enhanced lateral root branching (Xu et al., 2021).

In arabidopsis, the *WUSCHEL*-related homeobox (*WOX*) genes *WOX11* and *WOX12* are activated by auxin during the first step of *de novo* root organogenesis (Hu and Xu, 2016). After wounding, auxin accumulation activates the expression of these two *WOX* genes that promote the fate transition from regeneration-competent cells to root founder cells (Liu et al., 2014c), which activate the transcription of *WOX5* and *WOX7* required for the transition from root founder cells to root primordium (Hu and Xu, 2016). *WOX11* and *12* act redundantly to activate *LBD16/29* which are required for adventitious rooting (Hu and Xu, 2016). In *Populus*, several studies have demonstrated that *WOX* genes are also implicated in AR formation, suggesting a common role of *WOX* genes in this process (Figure 1). In *Populus tomentosa*, *PtoWOX5s* and *PtoWOX11/12s* play a major role in root development, and their expression was found to be strongly induced during the regeneration of ARs (Liu et al., 2014a). Li et al. (2018) and Xu et al. (2015) confirmed the involvement of *PtoWOX5a* and *PeWOX11a/b* in adventitious rooting in poplar. They showed that the overexpression of *PtoWOX5a* in *Populus tomentosa* promoted AR formation by increasing the number of roots, but inhibited AR elongation, whereas the overexpression of *PeWOX11a* or *PeWOX11b* in *Populus euphratica* increased the number of ARs and induced formation of ectopic roots in the aerial parts of the transgenic poplars. *PtoWOX5a* might control root development through the regulation of D-type cyclin (*CYCD*) genes which are involved in cell division and differentiation (Li et al., 2018). Since *PtoWOX5a* was expressed mainly in the root tip during callus formation step and AR emergence, it might indicate that *WOX5a* acts in *Populus* at a later stage of root organogenesis than *WOX11*, which is similar to arabidopsis. Nevertheless, in the *PtoWOX5a*-OE lines *WOX11* was upregulated suggesting that both *WOX5* and *11* are involved in AR development at the same time and that their relationship might be more complex (Li et al., 2018). The overexpression of another member of the *WOX* family, *PtoWUSa*, increased the number of ARs (Figure 1) but decreased their length (Li et al., 2020). In this study it was suggested that *PtoWUSa* is involved in the regulation of polar auxin transport in *Populus* during adventitious rooting since the *PIN-FORMED* auxin transporter genes were downregulated in the roots of *PtoWUSa*-OE plants. In arabidopsis, *pin2* and *pin5* mutants present shorter roots (Müller et al., 1998; Mravec et al., 2009), which might explain the inhibition of root elongation in poplar.

Moreover, transcription factors from the GRAS family such as *SCARECROW* (*SCR*) were also shown to be involved in AR development in trees. In arabidopsis, the *SCR* gene is involved in the organization of the root meristem (Di Laurenzio et al., 1996) and is required for distal specification of the quiescent center therein (Sabatini et al., 2003). In the forest species *Pinus radiata* and *Castanea sativa*, two *SCR-LIKE* genes have been shown to be induced by exogenous auxin and to play a role during the initial stages of AR formation (Sánchez et al., 2007). Similarly, the Pe*SCR* gene is involved in the adventitious rooting process in the hybrid poplar *P. deltoides* × *P. euramericana* and it interacts with the *SHORT-ROOT* (*SHR*) transcription factor *PeSHR* (Xuan et al., 2014; Figure 1). Finally, other genes related to auxin signaling have been identified during AR formation in poplar cuttings. The gene *PtaERF003*, a member of the *APETALA2/ETHYLENE RESPONSE FACTOR* (*AP2/ERF*) family, was found to be induced by auxin and to positively control rooting of the hybrid *P. tremula* × *P. alba* cuttings (Trupiano et al., 2013).

Auxin is the master regulator of adventitious rooting in different species including *Populus* and, so far, the regulatory model described for arabidopsis (Gutierrez et al., 2012; Lakehal et al., 2019) seems to be conserved, at least partially, in the *Populus* genus and within its taxonomic groups, although some differences have been observed. The fact that some genes present a distinct role in AR formation between the two species might indicate that they evolved with distinct functions and more studies are needed in order to define a regulatory scheme for *Populus* AR formation which could serve as a model for other tree species.

#### Jasmonate

Jasmonate (JA) participates in the regulation of several physiological processes and is especially important in the response to biotic and abiotic stresses (Bhatla, 2018b). The role of JA in AR formation it is not very clear yet and could be species-dependent according to Lakehal et al. (2020b). It has been shown that JA is a negative regulator of AR initiation in arabidopsis intact hypocotyls where *ARF*s and *GH3*s regulate the level of the active form of JA, jasmonoyl-isoleucine, which inhibits adventitious rooting through the COI1-MYC2-dependent pathway (Gutierrez et al., 2012). In contrast, after wounding, JA production is induced and activates *ETHYLENE RESPONSE FACTOR109* (*ERF109*), which induces auxin biosynthesis thereby promoting ARs in arabidopsis leaf explants. *ERF109* is later inhibited by JASMONATE-ZIM DOMAIN (JAZ) proteins to prevent hypersensitivity to wounding (Zhang G. et al., 2019). A sharp peak in JA is observed after cutting and prior to rooting, at the base of petunia, tobacco (*Nicotiana tabacum*) and pea (*Pisum sativum*) stem cuttings, suggesting that JA could be a positive regulator of AR formation after wounding in these species (Ahkami et al., 2009). Nevertheless, exogenous application of JA does not support this hypothesis since diverse responses with respect to adventitious rooting have been observed. In petunia cuttings, JA treatment inhibited AR formation in a dose-dependent manner. Lower JA concentrations (0.1–1.0 μM) did not change AR numbers, whereas higher concentrations (10 μM) significantly reduced the root number, and 100 μM JA completely inhibited AR formation (Lischweski et al., 2015). In arabidopsis, a low concentration of Methyl-JA (MeJA) treatment enhanced AR formation when combined with IBA and kinetin, a type of cytokinin, in dark-grown seedlings and in epidermis thin cell layers cultured *in vitro* (Fattorini et al., 2018). It was also demonstrated that the ability to regenerate ARs in the presence of exogenous JA involved crosstalk with ethylene through the *ETHYLENE INSENSITIVE3/ETHYLENE INSENSITIVE3-LIKE1* (*EIN3/EIL1)* signaling pathway (Fattorini et al., 2018).

Recently, Wei et al. (2020) showed that the gene *PuHox52*, from the HD-Zip I transcription factor family, is a positive regulator of AR development downstream of JA in *Populus ussuriensis* (Figure 1). *PuHox52* was consistently upregulated at different time points after cutting, and its overexpression significantly enhanced the number of ARs, whereas in RNAi transgenic lines the number of ARs decreased, thus confirming its role in promoting AR formation. *PuHOX52* gene expression was induced by exogenously applied MeJA, but was significantly reduced when treated with a JA biosynthesis inhibitor, confirming its role in AR development downstream of CO1-mediated JA signaling. Wei et al. (2020) also showed that *PuMYC2* was a target gene of *PuHox52*, and *PuMYC2*-*OE* lines showed a significantly higher number of ARs, whereas *PuMYC*2-*SRDX* lines produced fewer ARs than the wild type. These results show that *MYC2* acts as a positive regulator in AR formation in *Populus ussuriensis*. In contrast, At*MYC2* was shown to negatively regulate AR formation by activating CK signaling (Lakehal et al., 2020a). These contrasting results between arabidopsis and *Populus* indicate that *MYC* genes may have evolved with distinct functions. Since there are six *MYC2* paralogs in the poplar genome, some of them may have a specific role in *Populus*, compared to arabidopsis. It would be of interest to evaluate whether all these six *MYC* paralogs present the same promoting effect on adventitious rooting in *Populus*, and if there are differences between taxonomic groups. Moreover, *AtMYC2* acts as a negative regulator in arabidopsis intact hypocotyls, while *PuMYC2* activates adventitious rooting in *Populus* after wounding. It might be possible that wounding triggers other transcription factors that interact with *MYC2* promoting the rooting process in cuttings.

#### Cytokinins

Cytokinins (CKs) are plant hormones that promote cell division and are involved in different processes of plant growth and development. They act as antagonists of auxin in many aspects of development. For example, a high auxin/CK ratio induces root formation, whereas a low ratio stimulates the formation of shoots. Thus, CKs are known to inhibit adventitious rooting. Bollmark and Eliasson (1986) showed that CKs inhibit the differentiation of primordia at an early stage in root development. In a more recent study, Bustillo-Avendaño et al. (2018) suggested that CKs act first as positive activators of vasculature cell division and micro-calli formation, but in a later phase they act as negative regulators of root founder cell specification and root primordia initiation.

In the basal part of *Populus* cuttings, De Klerk et al. (1999) found opposite patterns in the auxin and CK concentrations during the first steps of adventitious rooting, suggesting that they play opposite roles in this process. Similar results were obtained in petunia and pea, in which, after wounding, auxin levels peaked rapidly, whereas CK content sharply decreased after roots were removed (Ahkami et al., 2013; Rasmussen et al., 2015). Ramirez-Carvajal et al. (2009) showed that the overexpression of a cytokinin type-B response regulator (*PtRR13*), which acts in the CK signaling pathway, reduced AR formation in cuttings of the hybrid *P. tremula* × *P. alba* (Figure 1) and stimulated the transcription of a negative regulator of vascularization (*COV1*) and a *PLEIOTROPIC DRUG RESISTANCE TRANSPORTER9* (*PDR9)*, an auxin efflux transporter, whereas it inhibited the transcript expression of two *AP2/ERF* genes TINY-like. In the proposed model CK signaling is reduced after shoot excision at the base of the cuttings, which enables coordinated expression of ethylene, auxin, and vascularization pathways leading to AR development. Moreover, in *Populus tomentosa*, the overexpression of *CKX2*, a cytokinin oxidase that converts active CKs to the inactive form, in the roots enhanced root growth without modifying shoot growth (Li et al., 2019), suggesting a negative role of CKs in rooting. In the *PtoWOX5a*-overexpressing plants genes from the CK signaling were affected, suggesting a crosstalk between auxin-inducible *WOX* genes and CKs.

These results are in agreement with recent findings that demonstrated that CKs act downstream of JA to inhibit AR initiation in arabidopsis. An *AP2/ERF gene, ERF115*, is activated by JA through the COI1-signaling pathway and repress AR initiation by activating CK signaling, probably by inducing the expression of *IPT3*, a gene involved in CK biosynthesis (Lakehal et al., 2020a). It is still unclear whether the negative role of CKs is phase-dependent in *Populus*, and if the different phases of AR formation have a distinct sensitivity to these hormones. Since the main source of CKs is removed in the cuttings, it would be interesting to study the effect of exogenous CK applications on ARs during the different rooting phases in order to better assess the role of these hormones in AR development.

#### Ethylene

Ethylene (ET) is a gaseous plant hormone which regulates many physiological and developmental stages and mediates adaptive responses to biotic and abiotic stress factors (Bhatla, 2018a). In arabidopsis root development, ET is part of a signaling pathway that modulates cell division in the quiescent center in the stem cell niche during the postembryonic development of the root system (Ortega-Martinez et al., 2007). Ethylene is induced by wounding as soon as 3 h after cutting at the base of sunflower (*Helianthus annuus*) hypocotyl cuttings, where a peak of ET was observed that was correlated with AR formation (Liu and Reid, 1992). In the hybrid aspen *P. tremula* × *P. tremuloides*, a peak of ET was observed 24 h after cutting, before the peak of free IAA and root formation, suggesting that ET may be involved in the early phase of AR formation (Hausman, 1993). Ramirez-Carvajal et al. (2009) observed an increase in the transcript abundance of the aminocyclopropane-1-carboxylate (*ACC*) synthase and oxidase genes, at the base of cuttings of the *P. tremula* × *P. alba* hybrid, after shoot excision. These genes code for enzymes of the ET synthesis pathway, and their increased expression might explain the ET peak observed after wounding in the study by Hausman (1993). Comparable results were obtained in petunia cuttings, in which *ACC* synthase and oxidase genes were rapidly induced after cutting, suggesting that ET is required for early adventitious root induction (Druege et al., 2014). In a recent study, exogenous treatments with GABA (γ-Aminobutyric acid) reduced ET content at the base of cuttings of the hybrid poplar *P. alba* × *P. glandulosa*, which correlated with a delay in adventitious root formation. This result provides evidence for the important role of ethylene at an early stage of AR formation in poplar (Xie et al., 2020).

Although ET has been shown to be positively involved in the early phases of AR formation, the specific role of this hormone is still unclear. Activation-tagged lines of the hybrid poplar *P. tremula* × *P. alba* showed that *ETHYLENE RESPONSE 2* (*ETR2*) and the RING-type E3 ligase *XBAT32* genes play a role in AR development, since both over-expressing lines showed increased rooting (Trupiano et al., 2013). Interestingly, *ETR2* negatively regulates ethylene signaling (Hua and Meyerowitz, 1998), whereas *XBAT32* is involved in the degradation of ACS, the enzyme catalyzing the rate-limiting step in ET biosynthesis, and thereby reduces ethylene biosynthesis (Lyzenga et al., 2012). These results suggest that ET acts as a negative regulator of AR development in *Populus*.

Like JA, the role of ET in AR formation can be complex and species-dependent since treatments with either ET precursors or inhibitors have resulted in opposite responses depending on the species or the growth conditions. In petunia cuttings, the application of aminoethoxyvinylglycine (EVG), an inhibitor of *ACC* synthase, reduced the number of ARs, whereas ACC application enhanced the number of roots but reduced the average root length (Druege et al., 2014). Similar results were obtained in tomato (*Solanum lycopersicum*) seedlings where ACC treatment increased AR development (Negi et al., 2010). Ethylene precursor ACC has also been shown to increase the number of ARs in stem cuttings of Norway spruce (*Picea abies*) (Bollmark and Eliasson, 1990), but a similar treatment reduced AR development in sunflower (Liu and Reid, 1992).

In *Eucalyptus* cuttings, at high auxin levels ET inhibits AR formation (Kilkenny et al., 2012), whereas in *Populus*, Ramirez-Carvajal et al. (2009) showed that the overexpression of a cytokinin response regulator (*PtRR13*) repressed AR development (Figure 1) and proposed a model which integrates ET, auxin and CKs pathways during AR formation. Rigal et al. (2012) showed that the gene *PtAIL1*, member of the *AP2/ERF* family similar to the arabidopsis *AINTEGUMENTA*, has a role in the control of adventitious rooting in *P. trichocarpa*, suggesting that members of the *AP2/ERF* family are involved in the rooting of poplar cuttings. Subsequently, Yordanov et al. (2017) showed that *BIG LEAF* (*BL*), the *Populus* ortholog of *A. thaliana STERILE APETALA* (*SAP*) belonging to the AP2-domain transcription factor family, is a positive regulator of AR development. *BL* gene expression was correlated with *PtAIL1* transcript abundance, suggesting that the rooting-inducing effects of *BL* could be mediated *via* modulation of *PtAIL1* expression.

These studies demonstrate the significant role of ethylene in AR formation in *Populus*, as well as its interaction with other plant hormones in this process. More studies are needed in order to confirm the positive effect of ET during the induction phase and the negative effect of ET in later rooting phases in *Populus*.

#### Gibberellins

The role of gibberellins (GAs) in AR formation is still unclear but a few studies have demonstrated that exogenous applications of gibberellic acid are inhibitors of AR formation in *Populus*. In the aspen *P. tremula*, the application of paclobutrazol (PBZ), an inhibitor of GA biosynthesis, was found to promote AR formation, whereas GA treatment had a negative effect on AR development in *in vitro* culture cuttings (Žiauka and Kuusiene, 2010; Vaičiukynė et al., 2019). Moreover, transgenic hybrid aspen *P. tremula* × *P. tremuloides* overexpressing *GA20ox1*, one of the genes that catalyzes the final step in the synthesis of bioactive GAs, had poor rooting efficiency, whereas overexpression of the *GIBERELLINS INSENSITIVE* (*GAI*) gene, which is a repressor of GA signaling, increased the number of ARs (Eriksson et al., 2000; Busov et al., 2006; Mauriat et al., 2014). Similarly, Busov et al. (2006) demonstrated that root growth of *in vitro* cuttings of the transgenic hybrid aspen *P. tremula* × *P. alba* lines overexpressing *GAI* or *REPRESSOR OF GAI-LIKE1* (*RGL1*) was increased two to three times compared to the control line. Exogenous GA application could not inhibit AR formation in these transgenic lines as it did in the wild type. Gou et al. (2010) showed that the gibberellin deficient *35S:PcGA2ox1* and the insensitive *35S:RGL1* transgenic poplars had increased lateral root proliferation and elongation under *in vitro* and greenhouse conditions, and that these effects were reversed by exogenous GA treatment.

Overall, these results show that GAs act as negative regulators of AR formation in *Populus*. Nevertheless, more studies are needed in order to determine the developmental stage at which GAs are detrimental for AR development. Indeed, *GA20ox1-OE aspen* was found to exhibit poor rooting that affected plant survival, but those plants that produced roots had the same total root biomass as the wild type at later developmental stages. This suggests that the effect of GAs varies with the root development stage (Eriksson et al., 2000). Moreover, Mauriat et al. (2014) suggested that the GA inhibitory effect in hybrid aspen was mediated by the perturbation of the polar auxin efflux, rather than the auxin or strigolactone signaling pathways. It is, therefore, important to elucidate the interacting role between GAs and other hormones in the adventitious rooting of *Populus*.

#### Abscisic Acid

Abscisic acid (ABA) is a plant hormone that helps plants to adapt to stresses. ABA is often accumulated under water stress and inhibits cell cycle progression. In flooded rice (*Oryza sativa*) ABA has been shown to negatively control AR emergence (Steffens et al., 2006). The tomato mutant isoline *sitiens*, affected in the gene responsible for the conversion of abscisic aldehyde to ABA, has a significantly increased number of ARs compared to the wild type (McAdam et al., 2016). In grape, leaf application of an inhibitor of ABA catabolism increased the percentage of rooted cuttings, and the endogenous level of IAA in leaves and the basal portion of cuttings subject to dehydration was also enhanced. Moreover, gene expression of *ARF6* and *ARF8*, positive regulators of adventitious rooting, was increased, whereas the expression of *ARF17*, a negative regulator of adventitious rooting, was significantly lower at the base of the stem cuttings (Tomiyama et al., 2020). In *Populus*, Vaičiukynė et al. (2019) measured the content of ABA in *in vitro* cultured shoots of two aspens (*P. tremula*, and *P. tremuloides* × *P. tremula*) with different rooting abilities. No differences in ABA content were found between the two genotypes, suggesting that the endogenous content of ABA in the shoots is not correlated with the rooting ability of these two genotypes. Nevertheless, treatment with ABA added to the medium reduced the number of roots in both genotypes, suggesting that exogenous application of ABA negatively affects adventitious rooting in *Populus*.

#### Brassinosteroids

Brassinosteroids (BRs) are plant steroid hormones that play a role in various developmental processes such as cell elongation and division, as well as in biotic and abiotic stress responses (Nolan et al., 2020). The effect of BRs on adventitious rooting is not yet well established. While in some species BRs at low concentrations have been shown to inhibit root development in cuttings (Roddick and Guan, 1991), treatment of Norway spruce cuttings with (22S,23S)-28-homobrassinolide (SSHB), a synthetic brassinosteroid, improved their rooting capacity (Rönsch et al., 1993). BRs may also interact with other plant hormones to modulate rooting. In arabidopsis it has been shown that BRs act as positive regulators of AR initiation and that they interact with auxin (Maharjan et al., 2014). In the *Populus* transgenic line overexpressing a cytokinin type-B response regulator (*PtRR13*), the *Brassinosteroid Enhanced Expression* (*BEE*) gene was in the top five most significantly up-regulated transcripts 24 h after cutting, suggesting that this gene is affected by the overexpression of *PtRR13*, and that *BEE* may also be involved in the negative regulation of adventitious rooting in this transgenic line (Ramirez-Carvajal et al., 2009).

#### Strigolactones

Strigolactones (SLs) are plant hormones derived from the carotenoid pathway. They participate in different physiological aspects of plant development such as apical dominance and root growth (Bhatla, 2018c). SLs are produced mainly in the roots and, even though in de-rooted cuttings the main source of SLs has been removed, these hormones still play a role in AR formation.

Exogenous applications of SLs have revealed species-dependent responses. In arabidopsis, the application of GR24, a synthetic SLs analog, suppressed AR formation in a dose dependent manner in both the wild type and the *max4* mutant, which has reduced SL synthesis (Rasmussen et al., 2012). In tomato cuttings, exogenous application of GR24 reduced the number of ARs, but a combination of GR24 and IAA restored the rooting to untreated levels (Kohlen et al., 2012). In contrast, in rice, the application of GR24 increased the number of ARs in the mutant SL-deficient *d10* (Sun et al., 2015). It has been observed that SLs interact with other plant hormones such as CKs and auxin during AR formation. Rasmussen et al. (2012) suggested that SLs and CKs act independently to inhibit AR formation, and that SLs negatively regulate auxin basipetal transport and accumulation in the rooting zone.

These results have been corroborated by studies of mutants or transgenic lines. In arabidopsis and pea, SL-deficient or response mutants exhibited enhanced adventitious rooting (Rasmussen et al., 2012). Similarly, in the hybrid *Populus* × *canescens*, the downregulation by an artificial miRNA of *MAX4*, a key gene in SL biosynthesis, increased significantly the number of ARs (Muhr et al., 2016) suggesting that SLs act as negative regulators of AR in arabidopsis and in *Populus*. In both the poplar *MAX4* knockdown and transgenic tomato cuttings with reduced SL content, the polar auxin transport from the apical tissues to the base of the stem cuttings was increased (Kohlen et al., 2012; Muhr et al., 2016). Contrasting results were obtained with rice, for which SLs seem to promote crown root formation by modulating auxin transport. SL-deficient (*d10*) and SL-insensitive (*d3*) rice mutants exhibited reduced AR production compared to the wild type (Sun et al., 2015).

Even though the exact role of SLs in AR formation is not clear yet, effects of these hormones on AR formation have been observed in various species. In *Populus* it seems that SLs act as negative regulators, but more studies need to be performed to confirm their role in adventitious rooting.

#### Other Molecules

A positive correlation between polyamine accumulation and the initial stage of adventitious rooting in *Populus* has been shown (Hausman et al., 1995), but contrasting effects could be observed depending on the polyamine. Putrescine has been reported to promote AR formation in the hybrid aspen *P. tremula* × *P. tremuloides* in media without supplemented auxin, whereas other polyamines, such as spermidine and spermine, inhibited root formation when combined with auxin (Hausman et al., 1994). Similar results were observed in apple rootstock shoots raised *in vitro* where putrescine enhanced rooting in the absence of IBA (Naija et al., 2009).

In a recent study, the effect of γ-Aminobutyric acid (GABA) on AR formation in the hybrid poplar *P. alba* × *P. glandulosa* was evaluated. Endogenous GABA accumulation in the *PagGAD2* overexpressing line, a gene encoding a glutamate decarboxylase necessary to form GABA, inhibited AR number and length. The effect of exogenously applied GABA was dose-dependent. Lower concentrations increased AR development, whereas higher concentrations inhibited it. When vigabatrin (VGB), an inhibitor of GABA degradation, was applied, AR growth decreased. With both GABA at the highest concentrations and VGB applications, endogenous GABA levels were increased, whereas in the lower GABA treatments the endogenous content decreased. These results provide evidence that endogenous GABA accumulation is negatively correlated with AR formation in poplar (Xie et al., 2020).

The role of phenolic compounds in adventitious rooting has long been known. They have been shown to either synergize or antagonize auxin action (Gorter, 1962; Basu et al., 1969). In different clones of *P. alba* and *P. canescens*, the external application of phenolic substances such as pyrogallol or salicylic acid improved rooting. This beneficial effect was greater when the natural ability to form roots was low. Moreover, addition of these compounds enhanced the positive effect of auxin (Bojarczuk and Jankierwicz, 1975). Pal and Nanda (1981) found similar results with catechol, which promoted AR formation on etiolated stem cuttings of *P. robusta*, in the presence of exogenous IAA. Nevertheless, while there was no difference when cuttings were kept at 25 or 35°C in the presence of auxin alone, the synergic effect of catechol was temperature- and concentration-dependent. At 35°C catechol became inhibitory when used at a concentration of 5 mg/l and above (Pal and Nanda, 1981). It was suggested that phenolic compounds induced rooting by reducing IAA decarboxylation and that their promoting effect occurs during the initial phase of rooting, similar to auxin (De Klerk et al., 2011). Flavonoids, a main class of phenolic compounds, can modulate auxin transport by either interacting with PIN2 or affecting the distribution of PIN proteins (Buer et al., 2010), and different flavonoids have effects of different magnitudes (Buer et al., 2013). Phenolics can also modulate the levels of reactive oxygen species (ROS) and buffer ROS accumulation that occurs when local IAA increases (Peer et al., 2013). Auxin catabolism involves oxidative decarboxylation by peroxidases, and since phenolics also modulate peroxidase activity, this may prevent auxin degradation at the base of cuttings (Fogacça and Fett-Neto, 2005; De Klerk et al., 2011). In mung bean (*Phaseolus radiatus* L), salicylic acid, a phenolic acid involved in biotic and abiotic stress responses, promotes AR formation *via* the process of hydrogen peroxide (H_2_O_2_) accumulation (Yang et al., 2013). H_2_O_2_ is one of the major members of the ROSs and plays essential roles as a beneficial signaling agent. It has been reported that H_2_O_2_ regulates lateral root formation (Chen et al., 2018) and is involved in AR formation in different plant species such as mung bean (Li et al., 2017) and marigold (*Tagetes erecta* L) (Liao et al., 2012). Recently it has been shown that genes involved in H_2_O_2_ homeostasis are overexpressed and clustered together at the base of poplar cuttings 8 days after cutting and that this peak in gene expression is correlated with the accumulation of H_2_O_2_ (Zhang Y. et al., 2019). In addition, exogenous application of H_2_O_2_ to these poplar cuttings increased the number of ARs, whereas dimethylthiourea (DMTU), a chemical H_2_O_2_ scavenger, had the opposite effect (Zhang Y. et al., 2019).

Peroxidases are a group of enzymes that catalyze the oxidation of a substrate by H_2_O_2_ or other organic peroxides. Many studies have shown a correlation between peroxidase enzyme activity and the rooting process, and it has even been proposed that changes in peroxidase activity could be used as biochemical markers for rooting phases in several species (Hand, 1994; Rout et al., 2000; Saxena et al., 2000; Hatzilazarou et al., 2006). During the course of rooting after wounding, an inverse relationship between endogenous auxin content and peroxidase activity has been highlighted (Gaspar et al., 1992; Nag et al., 2013). During the induction of ARs in cuttings in response to wounding, there is an increase in phenolic compounds, jasmonic acid and auxin at the base of the cuttings, with a lower peroxidase activity. On the other hand, during the initiation phase, peroxidase activity reaches its maximum level and auxin content decreases (Gaspar et al., 1992; De Klerk et al., 1999; Schwambach et al., 2008; Ahkami et al., 2009). Among the peroxidase isoenzymes, some of them can catalyze the oxidation of IAA, the so-called IAA-oxidases (IAA-Os). These IAA-Os regulate the IAA content and promote rooting after the induction phase. Güneş (2000) measured the enzymatic activity of IAA-Os during the rooting period of cuttings from three different *Populus* species, *P. alba*, *P. nigra* and *P. tremula*, and found a positive correlation between the enzymatic activity and the rooting ability of the cuttings. Both *P. alba* and *P. nigra*, had an increased IAA-O activity 8 days after cutting, in accordance with the root primordia formation, whereas in the aspen *P. tremula*, no roots were formed, and no IAA-O activity was detected. Similar results were observed in petunia cuttings, in which a higher peroxidase activity was observed after the induction phase during AR formation (Ahkami et al., 2013). This could be associated with the degradation of auxin *via* oxidative decarboxylation, necessary to reduce the amount of auxin in later stages of AR formation (Ahkami et al., 2013). In contrast, in *Eucalyptus globulus*, peroxidase activity increased with the loss of rooting capacity in older cuttings, while a decrease in IAA content was observed (Aumond et al., 2017). Nevertheless, additional experiments are required to confirm that this could be due to IAA-oxidase activity.

These results show that peroxidases and H_2_O_2_ are related to AR formation in *Populus*. Since it has been suggested that changes in peroxidase activity could be used as markers to detect good and bad rooting genotypes at an early rooting stage, it would be interesting to study in more detail the role of these agents in adventitious rooting. Moreover, in a study where rooting and non-rooting cuttings from *Ebenus cretica* genotypes were used, a fast-migrating soluble anionic peroxidase isoform was detected in the rooting genotype whereas it was absent from the non-rooting genotype, suggesting a specific role of this class of peroxidase (Syros et al., 2004). It is, therefore, important to identify the role and class of peroxidases that might be related to AR formation in *Populus* in the different taxonomic groups.

## Environmental Factors

### Biotic Factors: Symbioses Between Plant Roots and Microorganisms

Plant roots interact with soil microorganisms and this stimulates plant growth and nutrition. This beneficial relationship can also improve rooting and, therefore, the survival and yield of cuttings. Arbuscular mycorrhizae are an example of a symbiosis between plant roots and different fungal species which, as shown for different plants, can increase root branching (Péret et al., 2009). In poplar cuttings, inoculation with mycorrhizae significantly altered the root system, with an increase in the number and length of lateral roots (Hooker et al., 1992). Similar results have been obtained in hybrid aspen (*P. tremula* × *P. alba*) cuttings, where an ectomycorrhizal fungus stimulated branching of the adventitious roots (Felten et al., 2009). A model of fungus-induced auxin accumulation at the root apex which involves PIN9-dependent auxin redistribution together with IAA-based auxin signaling has been proposed (Felten et al., 2009). Moreover, a co-culture of *Populus* × *canescens* rooted cuttings with mycorrhizae increased plant survival and root biomass (Müller et al., 2013). Endophytic bacteria have also shown a positive effect on rooting of poplar cuttings. Hybrid poplar *P. deltoides* × *P. nigra* hardwood cuttings inoculated with different strains of bacteria presented significantly more roots after 10 weeks of growth (Taghavi et al., 2009).

Based on the results of these studies, the use of microbial inoculum seems promising for *Populus* agroforestry to enhance rooting, especially in difficult-to-root genotypes. Nonetheless, there is still limited field application of this technique due to interactions between plant genotype and different microorganisms, among other things (Zavattieri et al., 2016). More studies aimed at identifying suitable combinations between plant genotype and microorganisms, as well as the mechanisms underlying this interaction should be carried out.

### Light

Adventitious root formation is influenced by both light intensity and quality applied to the donor plants and during the rhizogenesis process itself. Light has long been considered an important factor for vegetative propagation in woody species. Stenvall et al. (2005) showed that light had a negative effect on the rooting of hybrid aspen cuttings of *P. tremula* × *P. tremuloides*, and the average rooting percentage was lower in the light than in the dark, even though the time for rooting did not change between conditions. In this study, significant variation in rooting between clones was also observed. Etiolation of the mother plants has been found to improve significantly the rooting of cuttings in some tree species, including *Populus* (Eliasson and Brunes, 1980; Maynard and Bassuk, 1986). It has been suggested that light effect on AR formation may affect growth regulators such as auxin and cytokinins, as well as carbohydrate availability and distribution (Klopotek et al., 2016; de Almeida et al., 2017). Pal and Nanda (1981) found that treatment with auxin increased the rooting of *P. robusta* cuttings and this effect was even better when catechol and sucrose were added, and cuttings were kept in the dark.

Light quality has also been shown to influence AR formation in woody species. In the difficult-to-root *Eucalyptus globulus*, far-red radiation on mother plants grown on media lacking sucrose increased the percentage of rooted cuttings, whereas this effect was not observed in the easy-to-root genotype *E. grandis* (Ruedell et al., 2013). Moreover, far-red treated *Eucalyptus globulus* plants presented an increased carbohydrate content and rooted better (Ruedell et al., 2015). In spruce, constant red light had a positive effect on rooting initiation of de-rooted seedlings, possibly by repressing the accumulation of the negative regulators jasmonate and cytokinins (Alallaq et al., 2020). In eastern cottonwood (*P. deltoides*), the rooting of microcuttings grown in media with exogenous auxin (IBA), was better under fluorescent light than under light emitting diodes (LEDs) (Hoffman et al., 2016). To date there are no studies of *Populus* evaluating how light quality and treatment duration affect AR formation, nor which target, *i.e*., the donor plant or the cuttings themselves during the rooting period, should be treated with light to enhance rooting. It is important to evaluate these parameters in order to understand the complex relationship between light and rooting in *Populus* cuttings, as well as the interactions with other factors such as hormones since it has been shown that, for example, red/far-red ratio affects basipetal auxin transport (Morelli and Ruberti, 2002).

### Pre-planting Treatments

Pre-soaking cuttings in water is a pre-planting treatment that increases the success of rooting and enhances the survival and growth of the plants. Desrochers and Thomas (2003) showed that pre-soaking cuttings in water for 2 to 4 days improved rooting in four hybrid poplar clones with different genetic backgrounds from the sections Aigeiros and Tacamahaca. Hansen and Phipps (1983) and Phipps et al. (1983) also showed that warming and pre-soaking cuttings before planting had a positive effect on rooting of cuttings of different poplar hybrids. Subsequently, Puri and Thompson (2003) studied the influence of three levels of initial water content (dried, soaked and fresh) on the rooting capacity of different soil water potentials in stem cuttings of the hybrid *Populus* × *euramericana*. Their results showed that soil moisture as well as pre-soaking of cuttings had a positive effect on rooting. Water-stressed cuttings took longer to root and formed fewer roots, whereas pre-soaked cuttings rooted better, especially in drier soil.

Pre-planting treatments may also have negative effects. For example, bud removal has been shown to have a negative effect on AR development in *Populus* cuttings. Removing 50% or more buds from the cuttings of ten poplar clones significantly reduced the number of ARs, but had no effect on the total root dry mass (Wiese et al., 2006).

To date, the pre-planting treatments with a positive impact on AR formation in *Populus* have only been evaluated in poplars from sections Aigeiros and Tacamahaca, which generally root well. Identifying pre-planting treatments with a positive effect on rooting in sections with poor rooting performance would be of great value for breeding programs.

### Edaphic Factors

Edaphic factors such as soil temperature, soil moisture, soil structure, and soil aeration play a major role in governing the rooting of poplar cuttings. Successful rooting in *Populus* is obtained at a soil temperature above 10°C, whereas at lower soil temperatures root and shoot growth are inhibited (Landhausser, 2003). Zalesny et al. (2005) evaluated different rooting traits, such as root dry mass, number of roots and root length, in 21 *Populus* genotypes, belonging to five different genomic groups, grown at different soil temperatures. They concluded that at least 4 days at a minimum of 14°C, along with sufficiently dispersed precipitation, were needed in order to obtain above-average rooting traits from hardwood cuttings. It was also suggested that different root phases may occur at different temperature thresholds. In another study, the same authors found differences in soil temperature thresholds for root promotion among different *Populus* genotypes: warmer soils promoted rooting in cottonwood (*P. deltoides*) whereas cooler temperatures were more effective for rooting in the hybrids evaluated (Zalesny et al., 2004). Wan et al. (1999) studied the relationship between water flow and growth in the aspen *P. tremuloides* at different soil and air temperatures. They suggested that growth inhibition in cold soils result in a limited ability of roots to deliver water to the leaves when the air temperature increases. The use of artificial soil heaters, like heating pads or hot water tubes, gave successful results in terms of rooting. Heating nets were used to test the response to rooting of different hybrid aspens at different temperatures, and the results showed that higher soil temperatures increased the rooting percentage (Stenvall et al., 2005). Warming the basal ends of softwood cuttings of Carolina poplar (*P. × canadensis*) to 30°C in a controlled air temperature, had a positive impact on root growth as well (Shibuya et al., 2013). It was suggested that the positive effect of warming the base of cuttings was related to a temperature gradient created by the warm water in the base and the cool apical ends.

Soil moisture is another important factor for successful rooting in *Populus*. The number of roots per cutting and the percentage of rooted cuttings of *P. deltoides* increased with increasing soil moisture up to the saturation point (Allen and McComb, 1956). Guo et al. (2011) evaluated the effect of 30-day flooding on different morphological and physiological changes, including the ability to form ARs in 13 hybrid poplars from sections Tacamahaca and Aigeiros. Ten out of the 13 clones developed ARs and their number and length increased with the duration of the flooding treatment. There was also variation between clones in the number of days needed to form ARs, which allowed the grouping of clones into high and low flooding tolerance.

Regarding soil texture, medium soil textures such as sandy loam, are commonly recommended for poplars to grow well (Baker and Broadfoot, 1979). Nevertheless, in the study by Rogers et al. (2019), no significant correlation between the soil texture and the root-shoot ratio was found for 15 different poplar clones from sections Tacamahaca and Aigeiros. The tested soil textures were clay, clay loam and sandy loam, obtained from different landfill sites and the plants were grown in a greenhouse. Harfouche et al. (2007) found that *P. alba* cuttings produced more and longer ARs when the cuttings were planted in gravel compared to river sand, but the percentage of rooted cuttings was not significantly different between the two soils. In another study, Böhlenius et al. (2016) found that root growth increased when *Populus trichocarpa* cuttings were planted in sandy soils compared to clay or forest soils, but they also concluded that the general establishment of poplar cuttings was independent of the soil textures examined in their study. The results of these studies suggest that soil texture is not as crucial as soil humidity in AR formation and plant establishment, but loose soils with sufficient humidity may contribute to better root growth. Having a larger root system increases the ability of plants to capture water and withstand stronger winds, improving drought resistance and their effectiveness as windbreaks.

### Mineral Nutrition: Macro and Micronutrients

It is generally accepted that a high Carbon/Nitrogen (C/N) ratio favors rooting, whereas a low one increases shoot growth. Nevertheless, in some studies it has been shown that the number and length of roots are positively correlated with the initial total N content in the cuttings (Druege et al., 2019). Only considering C/N ratios may be unreliable for predicting the rooting ability of a genotype. Other factors such as the redistribution of N during rooting, the source of and the total N content in the cuttings should also be considered (Haissig, 1986). In the hybrid *P. trichocarpa* × *P. deltoides*, N supplied by urea application to the leaves was better remobilized for its use in the new roots (Dong et al., 2004), whereas in poplar seedlings fertilized with ammonium the root dry weight and length were significantly reduced (Qu et al., 2016). Similar results were obtained for *Eucalyptus globulus* where the source of nitrogen affected the number of roots and the best rooting response was obtained with nitrate (Schwambach et al., 2005).

Phosphorus (P) is another macronutrient that has been shown to affect AR formation in a variety of species such as maize (*Zea mays*), rice, and common bean (*Phaseolus vulgaris*). Under limiting P conditions, AR development was increased in these species (Cruz-Ramírez et al., 2009). Similar results were observed with some *Eucalyptus* clones, with the endogenous P content in the mother plant being negatively correlated with the rooting ability of cuttings (da Cunha et al., 2009). Moreover, the positive effect of low phosphorus content on the rooting of tomato cuttings was facilitated by the action of ethylene (Kim et al., 2008).

Potassium (K) has been shown to promote AR formation in cucumber (*Cucumis sativus*) and bean cuttings (Zhao et al., 1991). In rice grown in low K soil, the ectopic expression of *WOX11* in the roots increased K acquisition efficiency and root biomass (Chen et al., 2015).

Calcium (Ca) also plays an essential role in AR formation. In the absence of Ca in the media, only 55% of cuttings from the hybrid aspen *P. tremula* × *P. tremuloides* developed ARs and the addition of LaCl_3_, a calcium channel blocker, completely inhibited rooting in these cuttings (Bellamine et al., 1998). Recently, Xiao et al. (2020) showed that poplar cuttings grown on hydroponic solutions added with 1 mM of Cl_2_Ca significantly increased the AR length and fresh weight, whereas 6 mM had the opposite effect. These results confirm that Ca regulate late phases of AR formation in *Populus*. By transcriptomic analysis they identified a Ca sensor, *PdeCML23-1*, from the calmodulin-like protein family, and its overexpression promoted AR elongation in the hybrid poplar ‘NL895’ by inhibiting, probably, the transport of Ca into the cytoplasm

Of the micronutrients, excessive Zinc (Zn) content has a negative effect on rooting of *Populus alba* cuttings (Castiglione et al., 2007). In addition, iron (Fe) has been shown to be one of the most limiting factors in AR formation in petunia (Hilo et al., 2017). Because Fe is immobile in the plant, foliar applications of Fe did not have the same effect as those applied at the stem base. Fe is a key constituent of many enzymes and, therefore, its deficiency may affect many physiological processes. In *Eucalyptus*, Fe deficiency during the induction phase significantly increased the root length, whereas this effect was not observed if the deficiency occurred in the formation phase (Schwambach et al., 2005).

It is clear that the content of macro and microelements affects AR formation in different species. However, very few studies analyzing the individual and collective role of these nutrients have been carried out in *Populus*. A more complete study examining how mineral nutrition and hormonal balance interact may be beneficial to understand AR development in *Populus* cuttings.

## Concluding Remarks

Adventitious rooting is one of the most important traits in clonally propagated trees such as *Populus*. Plant establishment and survival, as well as adaptation to different environments is essential for breeding programs and they rely on a good rooting system. Different *Populus* genotypes present a broad variation in AR formation. Sections Aigeiros and Tacamahaca exhibit the best rooting performance and, therefore, are the most thoroughly studied and are normally used as elite parents. Nonetheless, traits from species belonging to other sections are desirable, such as the fiber quality from hybrid aspens, and introducing these traits by crossing is very limited. It is therefore important to study the factors and molecular bases that control AR formation in the different *Populus* genotypes in order to exploit the phenotypic diversity present in the genus.

**FIGURE 2:**
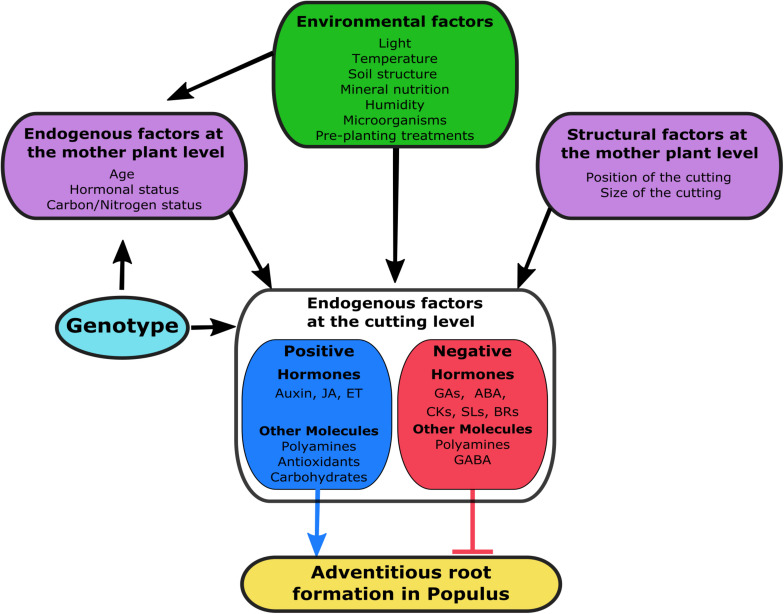
Factors affecting adventitious root formation in *Populus*. The genotype the mother plant will have an effect on the ability of the cutting to root. Microorganisms, soil mineral content, humidity and texture, light quality and intensity, soil and air temperature, as well as pre-planting treatments are the environmental factors that influence both the mother plant, from which the cutting is collected, and the rooting process in the cutting itself by affecting the endogenous factors. Mother plant status such as aging, physiological condition, as well as the size and position of the branch that is collected, and the date of branch collection, will influence the endogenous factors in the cutting. These endogenous factors will directly affect the rooting process in *Populus.* Auxin, Jasmonate (JA), Ethylene (ET), some polyamines and antioxidant compounds, such as salicylic acid and flavonoids, and carbohydrate content and distribution, which are highlighted in blue, are endogenous factors that positively regulate AR formation. In contrast, gibberelins (GAs), cytokinins (CKs), absicic acid (ABA), brassinosterods (BRs), strigolactones (SLs), γ-Aminobutyric acid (GABA), and some polyamines are negative regulators of AR formation in *Populus*, and are highlighted in red.

Adventitious root formation is a complex trait affected by many internal and external factors. Out of all of these, hormones seem, so far, to be the most important and central ones, as they not only interact with each other (Figure 1) but also with environmental cues (Figure 2). Most studies have focused on the role of auxin in AR formation in *Populus* which seems to be the master hormone regulator of this process, but complex crosstalk with and between other phytohormones, and also with external factors exists and this complexifies the mechanisms controlling adventitious rooting throughout the rooting process. Thus, more detailed studies are needed in which these interactions are taken into consideration and different genotypes are analyzed.

In recent years, many transcriptomic analyses performed during the different rooting phases have been published and many genes involved directly or indirectly in the rooting process have been identified. During the first hours after wounding, many genes change their expression profile, suggesting that they may play a role in the rooting process at a very early stage. Nevertheless, because of the great diversity in the rooting ability of various *Populus* genotypes, comparing results from different studies is very difficult and may not unravel the complex rooting process. It would be very interesting, though, to compare good and poor rooting genotypes in order to identify genes that are responsible for the differences observed. Performing transcriptomic analyses to compare extreme phenotypes could be a starting point to identify putative candidate genes that either induce or repress adventitious rooting in *Populus* species.

## Author Contributions

FB conceived the manuscript, prepared the figures, and wrote the manuscript. CB reviewed and edited the manuscript. Both authors approved the final manuscript.

## Conflict of Interest

The authors declare that the research was conducted in the absence of any commercial or financial relationships that could be construed as a potential conflict of interest.
